#  Curcumin Can be Acts as Effective agent for Prevent or Treatment of Alcohol-induced Toxicity in Hepatocytes: An Illustrated Mechanistic Review

**DOI:** 10.22037/ijpr.2020.112852.13985

**Published:** 2021

**Authors:** Elham Salehi, Mohammad Mashayekh, Fereshteh Taheri, Mina Gholami, Majid Motaghinejad, Sepideh Safari, Afrah Sepehr

**Affiliations:** a *Department of Pharmaceutical Chemistry, Faculty of Pharmaceutical Chemistry, Pharmaceutical Sciences Branch, Islamic Azad University (IUAPS), Tehran, Iran. *; b *Razi Drug Research Center, Iran University of Medical Sciences, Tehran, Iran. *; c *Department of Medicinal Chemistry, Faculty of Pharmacy, Tehran University of Medical Sciences, Tehran, Iran.*

**Keywords:** Curcumin, Alcohol, Oxidative stress, inflammation, Hepatocellular degeneration

## Abstract

Previous studies have shown that alcohol abuse can cause serious liver damage and cirrhosis. The main pathway for these types of hepatocellular cell neurodegeneration is mitochondrial dysfunction, which causes lipid peroxidation and dysfunction of the glutathione ring and the defect of antioxidant enzymes in alcoholic hepatic cells. Alcohol can also initiate malicious inflammatory pathways and trigger the initiation and activation of intestinal and extrinsic apoptosis pathways in hepatocellular tissues that lead to cirrhosis. Previous studies have shown that curcumin may inhibit lipid peroxidation, glutathione dysfunction and restore antioxidant enzymes. Curcumin also modulates inflammation and the production of alcohol-induced biomarkers. Curcumin has been shown to play a critical role in the survival of alcoholic hepatocellular tissue. It has been shown that curcumin can induce and trigger mitochondrial biogenesis and, by this mechanism, prevent the occurrence of both intrinsic and extrinsic apoptosis pathways in liver cells that have been impaired by alcohol. According to this mechanism, curcumin may protect hepatocellular tissue from alcohol-induced cell degeneration and may therefore survive alcoholic hepatocellular tissue. . Based on these mechanisms, the protective functions of curcumin against alcohol-induced cell degeneration due to oxidative stress, inflammation, and apoptosis events in hepatocellular tissue have been recorded. Hence, in this research, we have attempted to evaluate and analyze the main contribution mechanism of curcumin cell defense properties against alcohol-induced hepatocellular damage, according to previous experimental and clinical studies, and in this way we report findings from major studies.

## Introduction

**Figure 1:**
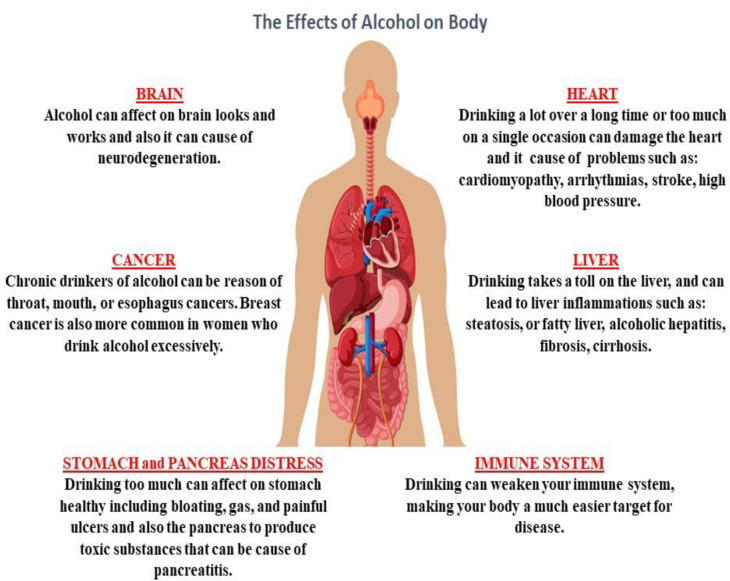
Negative effects of alcohol on body organs

Alcohol is a sedative-hypnotic agent which makes it a potential candidate for abuse because of its pharmacological properties for inducing sedation (1). Alcohol abuse has been on the rise in recent years (2). Studies have shown that alcohol can cause cell degeneration in multiple systems, such as the central nervous system (CNS), the cardiovascular system, the gastrointestinal system, the respiratory system, and the degeneration and disturbance of organs associated with these systems (Figure 1) (2-4). Alcohol abuse causes oxidative stress, apoptosis and inflammation in multiple organ cells, particularly in the liver (5-11).

**Figure 2:**
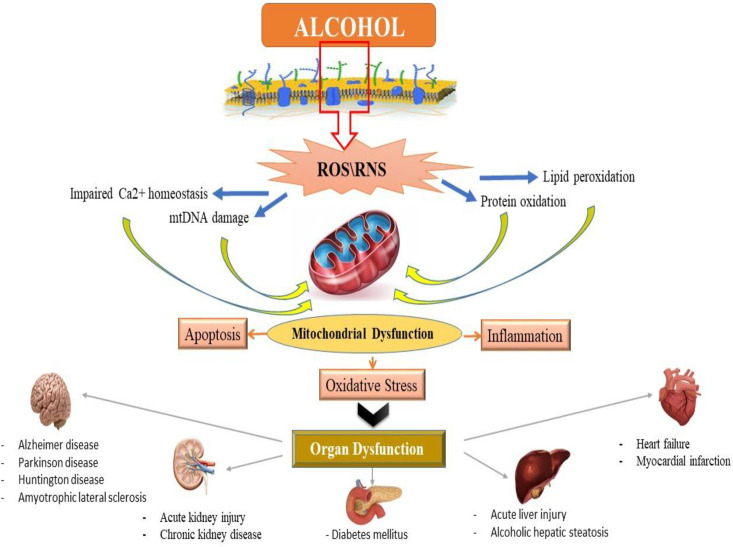
Alcohol-induced organ toxicity was mediated by mitochondrial dysfunction and the consequences of oxidative stress, inflammation and apoptosis induced by organ damage and dysfunction. ROS: Reactive Oxygen Species. RNS: Reactive Nitrogen Species

Alcohol exposure induces cell death signaling pathways and apoptosis in the liver and other organs and causes organ function disorders (11-13). Potential effects of alcohol on the induction of apoptotic proteins such as caspases-3, 9 and 8 and DNA fragmentation have been demonstrated in *in-vitro* and *in-vivo* studies (14, 15). Alcohol induced the release of cytochrome c and reduced mitochondrial viability (6, 15). Previous studies have shown that mitochondria play a key role in the regulation of alcohol-induced cell toxicity, especially neuro-apoptosis, and inducing oxidative stress and inflammation in neural cells (16-18). All of these studies have assumed that these types of increased inflammation, oxidative stress, apoptosis and mitochondrial alcohol dysfunction are responsible for its degenerative properties in multiple organs (Figure 2). On the other hand, in recent years, the use of new cell-protective compounds with therapeutic possibilities for the prevention or treatment of drug abuse of induced molecular and organ-functional complications has dramatically increased (19). 

**Figure 3.**
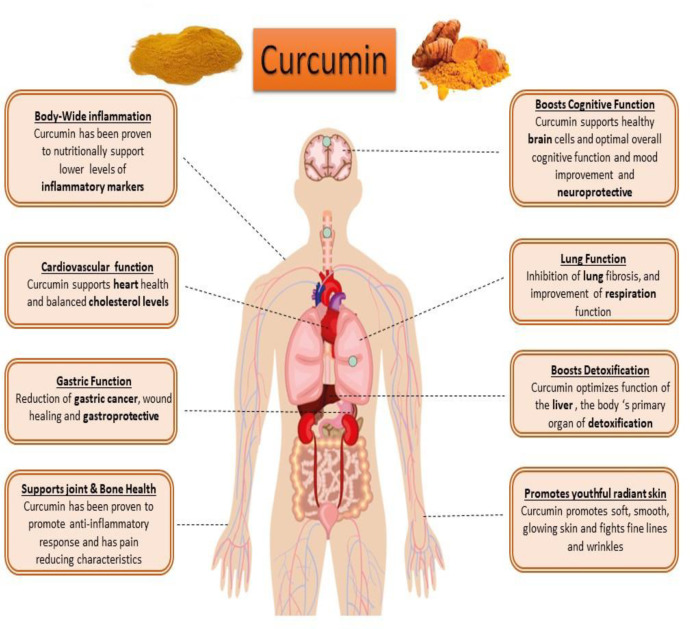
Protective role of curcumin in multiple body systems

Curcumin (diferuloyl methane) is the most abundant component of *turmeric* extracted from the Curcuma longa plant rhizomes (20, 21). This non-nutritive yellow pigment is an established nutraceutical dietary phenol and is therefore of great medicinal significance and pharmacological value in multiple body systems (Figure 3) (21).

**Figure 4:**
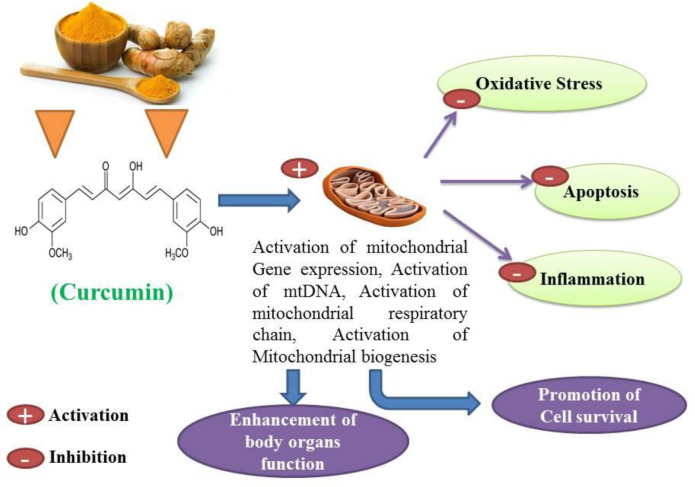
The protective effect of curcumin in multiple organs of the body has been mediated by mitochondrial biogenesis, which ultimately prevents oxidative stress, inflammation and apoptosis, contributing to the enhancement of body organ function

Curcumin has antioxidant, anti-inflammatory, anti-apoptotic, immunomodulatory and cell-protective properties (22-25). Curcumin has been proposed as a putative agent in the treatment of certain cell degenerative disorders. It may cause organ function modulation in some multiple disorders, and several studies have shown that apoptosis can be inhibited to some extent by the protective properties of curcumin (26, 27). The synergistic effects of curcumin on the activation of the antioxidant enzyme and mitochondrial biogenesis, along with its properties in inhibiting oxidative stress have also been investigated and published (28, 29). Studies have also shown that curcumin possesses antioxidant and anti-inflammatory properties that suppress major pro-inflammatory cytokines (30, 31). Studies have shown that curcumin anti-apoptotic effects and its properties on mitochondrial biogenesis can positively affect multiple organ cells (Figures 3 and 4) (26, 31). Several previous studies have suggested that curcumin may be a protective agent against alcohol-induced oxidative stress, inflammation apoptosis, mitochondrial dysfunction, and cell degeneration in hepatocellular tissue and has cell protection properties (6, 32-34). This review aims to provide a brief overview of the protective or inhibitory effects of curcumin in alcohol treatment caused by oxidative stress, inflammation, apoptosis, mitochondrial dysfunction and cell degeneration.

**Method of searching**

To find available data and information in the literature on the role of hepatoprotective properties of Curcumin in the prevention or treatment of hepatocellular alcohol-induced liver damage and oxidative stress, inflammation and apoptosis/cell death sequences, we searched multiple databases such as Web of Science, PubMed, Elsevier Science Direct, Google Scholar, Core Collection, Cochrane with the keywords Curcumin plus alcohol. For this review paper, all published documents on hepatoprotective properties of Curcumin against alcohol-induced hepatocellular damage during the years 1990-2019 were reviewed.

**Alcohol**

**Figure 5:**
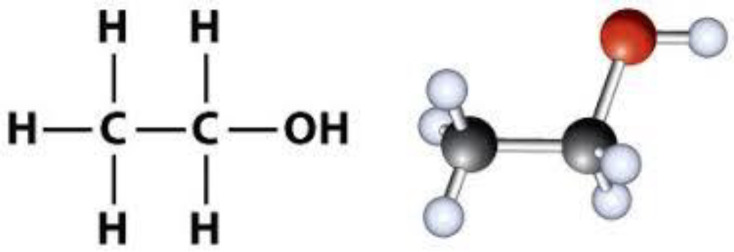
Structure of ethanol (Alcohol).

Alcohol is an organic compound in which the functional hydroxyl group (–OH) is bound to carbon (Figure 5). The term alcohol is initially referred to as primary ethanol (ethyl alcohol), which is used as a drug and is the main alcohol in alcoholic beverages. Due to its pharmacological properties, its consumption has recently increased (35, 36).

**Curcumin**

**Figure 6:**
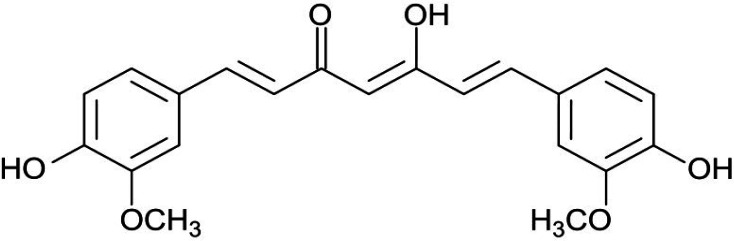
Structure of curcumin

Curcumin (a hydrophobic polyphenol) is the most important bioactive chemical constituent of *turmeric* (golden spice) (Figure 6). This compound was extracted from the herb rhizome Curcuma longa, which belongs to the family of Zingiberaceae. It has a wide variety of medicinal and pharmacological properties (21, 37 and 38). 

**Curcumin and alcohol-induced oxidative stress**

Previous studies have shown that chronic alcohol abuse leads to oxidative stress, mitochondrial dysfunction, respiratory enzyme alteration in hepatic cells. These studies have also shown that alcohol can induce oxidative stress in human and animal hepatocellular tissues (39-41). Alcohol consumption causes an increase in lipid peroxidation and an increase in MDA (Malondiadehyde) as a marker for lipid peroxidation. Such results are similar to previous reports showing that alcohol causes hepatic cell membrane damage (42, 43). Several results indicated that alcohol induces GSH (Glutathione) and GSSG (Glutathione disulfide) alterations in mitochondria, and chronic alcohol use may decrease GSH and increase GSSG levels in hepatic cells. These results confirm previous studies showing that alcohol increases the level of hepatotoxicity by converting glutathione in a reduced protective form (GSH) to oxidized damaging form (GSSG) (14, 43-46). The protective role of GSH against oxidative stress has also been extensively reported in the literature (45, 46). GSH can inhibit lipid peroxidation and act as an antioxidant mediator by scavenging free radicals (47, 48). Alcohol consumption induces the degradation of antioxidant enzymes in hepatic cells (48, 49). Treatment of animals and human subjects with alcohol decreases the activity of GPx (Glutathione Peroxidase) and GR (Glutathione Reductase) in the liver tissue. Chronic alcohol consumption in adult rats has been shown to reduce the production of antioxidant enzymes, such as GR and GPx, which play a role in the glutathione pathway (49-52). A similar previous study indicated that alcohol could reduce the activity of SOD (Superoxide Dismutase) in the liver (53, 54). Alcohol has been shown to decrease GR activity in the liver cells of animal subjects (49, 55 and 56). It has been shown that GR is the main enzyme responsible for converting glutathione from oxidized (GSH) to reduced form (GSSG) (55, 57). The antioxidant activity of this enzyme is mediated by increased GSH levels (47, 55). Several enzyme systems, including the cytochrome P450 (CYP450), 2E1-dependent microsomal monoxygenase system, the mitochondrial respiratory chain and the cytosolic enzymes xanthine oxidase and the aldehyde oxidases have been implicated as sources of superoxide anion (O^2 -^) and the hydroxyl radical (OH^-^) O^2-^ and Hydrogen Peroxide (H_2_O_2_) in parenchymal cells during ethanol intoxication (40, 58). Previous results indicate the contribution of intracellular and extracellular sources of Reactive Oxygens Species (ROS) to ethanol-induced oxidative stress (59, 60). Nonetheless, CYP2E1-produced ROS in liver tissue appears to be more effective in causing intracellular oxidative injury than ROS from activated phagocytes (60-62). Morphological and functional abnormalities of mitochondria are main causes of alcohol-induced hepatocyte injury (53, 54). As discussed above, ethanol stimulates ROS formation in mitochondria, leading to a decrease in mitochondrial GSH content that induces oxidative damage (8, 59, 63). Loss of mitochondrial GSH during chronic ethanol consumption causes inhibition of Adenosine Triphosphate (ATP) synthesis (62, 64). Ethanol-prompt lipid peroxidation was also associated with damage to mitochondrial oxidative-phosphorylation rings (39, 65 and 66). Mitochondria obtained from both acute and chronic alcohol-treated rats display oxidative changes in mitochondrial DNA and the efficacy of mitochondrial oxidation of nicotinamide Adenine Dinucleotide (NADH) has also decreased in hepatic cells of these subjects (18, 66). Mitochondrial DNA deletions have also occurred in alcohol-consuming subjects [64]. Induction of oxidative stress in liver mitochondria is associated with the collapse of mitochondrial membrane potential and the onset of mitochondrial permeability transition (MPT), which results in ion transport dysfunction and therefore cell damage (67, 68). Many previous studies have demonstrated the hepato-protective role of curcumin and have clarified its mechanism, and some studies have shown that curcumin by modulation of oxidative stress can inhibit alcohol-induced hepatotoxicity (23, 34). Curcumin as the key constituent of *turmeric* is known for its antioxidant and radical scavenging properties (20, 21). Curcumin enhances the systemic oxidative stress levels and increases the antioxidant balance activation of alcohol-induced hepatotoxicity in human and animal subjects (32, 69). Studies have shown that curcumin can effectively scavenge free radicals in alcohol by causing hepatic damage (70). Current evidence indicates that curcumin is mainly associated with decreased lipid peroxidation in alcohol-treated human and animal subjects (24, 71). Curcumin can inhibit alcohol-induced liver damage by scavenging these free radicals and inhibiting lipid peroxidation processes (24, 72). Curcumin, therefore, by possessing a high efficacy in preventing lipid peroxidation, may protect the hepatic cell membrane from alcohol-prompt oxidative damage (72, 73). In this scenario, curcumin therapy in animal models (multiple doses of 5, 10, 40 and 60 mg/kg) in alcohol-dependent rats may inhibit this alcohol-dependent effect and reduce the amount of MDA and lipid peroxidation in the liver tissue (74, 75). Previous studies have reported that curcumin has hepatoprotective effects; these findings have demonstrated the properties of curcumin mediated by inhibition of free radicals in the liver that are impaired by alcohol consumption (23, 32). Inconsistent with this issue, previous data have shown that different doses of curcumin may increase the GSH content and decrease the GSSG levels in animals receiving alcohol (64, 70, 76). Curcumin also neutralizes the harmful effects of alcohol on the glutathione ring (76, 77). Such findings have also been supported by previous studies showing that curcumin may increase the levels of glutathione in alcohol-dependent rats (55, 78). Some parts of the hapato-protection of curcumin against alcohol have been mediated by modulating the glutathione ring (69, 78). According to this study curcumin has also been shown to have potential effects on the regeneration of glutathione caused by chronic alcohol in vital organs, including the brain, liver and kidneys (79, 80). Previous studies have demonstrated the hepato-protective effects of curcumin mediated by the glutathione pathway (23, 81). It is noteworthy that curcumin has been shown to increase GSH and activate the glutathione pathway and decrease GSSG production in alcohol-treated subjects (72, 73 and 82). The antioxidant properties of curcumin in the modulation of the glutathione pathway against alcohol have been well understood and it has been shown that the appropriate balance between oxidant (GGSG) and antioxidant (GS) is a vital factor in the effective performance of the human liver metabolism system (24, 73, 82). In addition, curcumin has been shown to cause the preservation of mitochondrial biogenesis and antioxidant physiognomies in the strategic organ of alcohol-treated rats and human cases (83, 84). Curcumin causes an increase in the activity of SOD, GPx, catalase (CAT) and GR that stimulates antioxidant defenses in the liver of alcohol treated subjects (23, 25, 85). Curcumin has been shown to play a critical role in maintaining SOD and GPx function in liver cells and reversing alcohol-induced inhibition of CAT (23, 33, 86). These functions of curcumin may ultimately result in a reduction in lipid peroxidation and thus improve the harmful effects of alcohol in the liver (33, 86).

**Figure 7:**
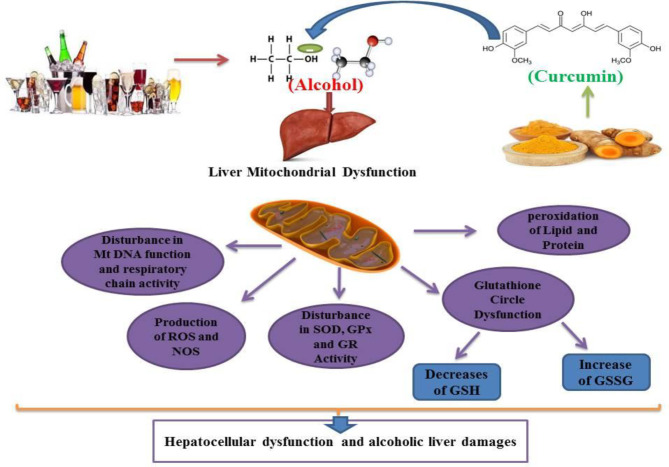
Curcumin can modulate alcohol-induced hepatocellular mitochondrial dysfunction, reduce or inhibit alcohol-induced lipid peroxidation, glutathione circulatory dysfunction, antioxidant enzyme (SOD, GPx and GR) activity dysfunction, and also inhibit alcohol-induced ROS and NOS formation. Curcumin attenuates alcohol sequels on mitochondrial respiratory chain and Mt DNA performances. GSH: Glutathione, GSSG: Glutathione disulfide, SOD: Superoxide Dismutase, GPx: Glutathione Peroxidase, GR: Glutathione Reductase, ROS: Reactive Oxygen Species. NOS: Nitrogen Oxygen Species. Mt DNA: mitochondrial DNA

 As shown, *in-vivo* and *in-vitro *studies have verified the impact of curcumin on the antioxidant defense system, lipid peroxidation levels, and ROS levels in hepatic cells of alcohol consumed subject (31, 33 and 76). Curcumin increases the conversion of GSSG to GSH by activating GR and, with this mechanism, protects haptic cells against alcohol-induced oxidative stress (24, 78). These studies have shown that curcumin has the potential for free radical scavenging and antioxidant enzyme activation in hepatic cells exposed to alcohol in animals or humans. Moreover, since oxidative stress is implicated in most alcohol-induced hepatic failure, studies have investigated the possible protective effect of curcumin as an antioxidant against this type of failure (Figure 7). 

**Curcumin and alcohol-induced inflammation**

Previous studies have shown that prolonged alcohol intake increases pro-inflammatory markers such as Tumor necrosis factor-alpha (TNF-α) and Interleukin 1 beta (IL-1β) in liver tissue. (87-89). The increase in pro-inflammatory markers has been shown to cause the degenerative effects of alcohol (89, 90). Relatively large bodies of previous studies have indicated that alcohol can stimulate cytokine pathways in liver cells, and this phenomenon causes hepatic cell degeneration. (9, 89, 91). The association between average daily intake of alcohol and C-reactive Protein (CRP) concentrations and other positive and negative markers of systemic inflammation in human subjects was investigated (45, 92 and 93). There is increasing evidence that inflammation plays a key role in hepatic cell fibrosis caused by chronic alcohol intake (94). Several previous studies have shown that alcohol consumption causes increased activation of various types of inflammatory cells, including monocytes, macrophages, T lymphocytes, mast cells, smooth muscle cells, and pro-inflammatory molecules such as cytokines and growth factors (11, 12, 94). According to the key concept of this research, the increase in pro-inflammatory biomarkers in the liver cell is a compensatory and protective response to malicious effects of alcohol (12, 95). On the other hand, some other studies claim that the controversial reports of these studies indicated that moderate alcohol consumption appears to inhibit the development of interleukin 6 or its action at hepatocytes (96). In contrast, high concentrations of interleukin 6 and other cytokines are found in alcoholic liver disease (96, 97). Ethanol or its metabolites may also modulate cytokine release from adipose tissue (97), an important source of basal interleukin 6 development and tumor necrosis factor (97, 98). According to these findings, mild to moderate alcohol abuse induces decreased inflammation, whereas chronic and high alcohol intake increases the development of pro-inflammatory biomarkers and triggers inflammatory cell reactions in hepatocellular tissue (39, 59). As described above, ethanol causes the formation of inflammatory and oxidative stress biomarkers in mitochondria and decrease in mitochondrial function which triggers hepatocellular damage (59, 99). The defeat of mitochondrial biogenesis during chronic ethanol drinking causes dysfunction of ATP synthesis (100, 101). Evene ethanol-prompt mitochondrial dysfunction results in liver tissue inflammatory response (97, 102, 103). Alcohol induced improvements in mitochondrial DNA and mitochondrial inflammatory biomarker efficacy have also increased in hepatic cells (39, 100 and 104). Alteration of mitochondrial DNA gene expression is responsible for the inflammatory process following alcohol consumption (100, 105). The incidence of inflammation in the mitochondria of the liver is correlated with the failure of the mitochondrial membrane potential (MMP) and the cause of the mitochondrial permeability transition (MPT) dysfunction resulting in hepatocellular collapse (100, 102 and 106). TNF-α is a major mediator of inflammation in alcohol-treated subjects. This effect is regulated by the activation of a transcription factor, nuclear factor kappa beta (NF kβ) (32, 97 and 107). NF kβ regulates most inflammatory mediators, such as chemokines and cytokines, adhesion molecules, kinases and enzymes (108). According to a previous study, alcohol-induced alteration of mitochondrial DNA gene expression is responsible for the production of NFkβ, which is the key factor in the initiation of the inflammatory process (17, 107 and 109). Curcumin has been shown to be potent hepato-protective and confirmed that curcumin can prevent alcohol-induced hepatotoxicity by modulating inflammatory events (24, 32 and 110). In addition, it has been shown that curcumin significantly decreased inflammatory markers in rats and human subjects receiving or consuming alcohol at different doses (24, 32, 75 and 111). Curcumin has a protective effect against inflammation; previous studies indicate that curcumin may suppress TNF-α and tumor growth factor beta 1 (TGF-β1) as well as decrease potential inflammation and liver injury (33, 73 and 112). Previous data suggest that some aspects of the anti-inflammatory action of curcumin have been mediated by inhibition of certain inflammatory cytokines and proteins (113, 114). A similar study suggested that curcumin has strong anti-inflammatory properties as well as a potential for repositioning, rendering it suitable for the treatment of autoimmune diseases, which is made possible by inhibiting inflammation and decreasing inflammatory markers (31, 86 and 114). The immune system was conceptually classified as an innate and adaptive subsystem (31, 86). Innate immunity provides a rapid response well before the evolution of antigen-specific responses provided by adaptive immunity (115, 116). Drinking or consuming alcohol has been shown to activate all immune system forms (117, 118). Curcumin can significantly affect both the innate and adaptive arms of immunity by altering immune cells such as macrophages, monocytes, natural killer cells (NK cells), T cells, and B cells (31, 32). The role of curcumin as a dose-and time-dependent decoration has been approved to reduce the expression of growth and survival of genes in inflammatory processes such as NFkβ in alcohol-treated subjects (119-121). The pathway through which curcumin may exert its suppressive effects on alcohol-induced inflammation has been suggested as an NFkβ signaling pathway. It has been observed that this compound reduces macrophage activation in hepatocellular tissue (31, 32, 119-121). Besides, it was shown that some parts of curcumin were mediated against alcohol-induced direct and indirect inflammatory by down-regulating the function of various signaling mediators, including down-regulation of Cyclooxygenase-2 (COX-2), mitogen-activated and Janus kinases, and inhibiting the generation of TNF-alpha, IL-1,IL -2,IL -6,IL -8, IL12, IL12, and 5′-Adenosine monophosphate-activated protein kinase (26, 32, 122 and 123). Curcumin also acts as an anti-inflammatory agent by inhibiting the generation of nitric oxide (NO) as well as the inducible expression in hepatocellular nitric oxide synthase (iNOS) (123, 124). The effect of curcumin in reducing inflammation is believed to be due to inhibition of the production of alcohol-induced TNF-α (32). Some parts of the anti-inflammatory effect of curcumin have been mediated by its protective effects in the mitochondria of the liver (32, 71 and 125). This agent activates and regulates mitochondrial membrane potential (MMP) and causes mitochondrial permeability transition biogenesis (MPT) activity which results in hepatocellular survival in inflammatory events such as alcohol administration (28, 32, 76, 125 and 126). All these findings suggest that curcumin possesses the potential for anti-inflammatory properties in alcohol-exposed animal or human hepatic cells. However, because inflammation is involved in most of the hepatic tissue fibrosis induced by alcohol; studies have investigated the potential protective effect of curcumin as an anti-inflammatory agent in inflamed liver induced by alcohol.

**Curcumin and alcohol-induced Apoptosis and Cell death**

**Figure 8:**
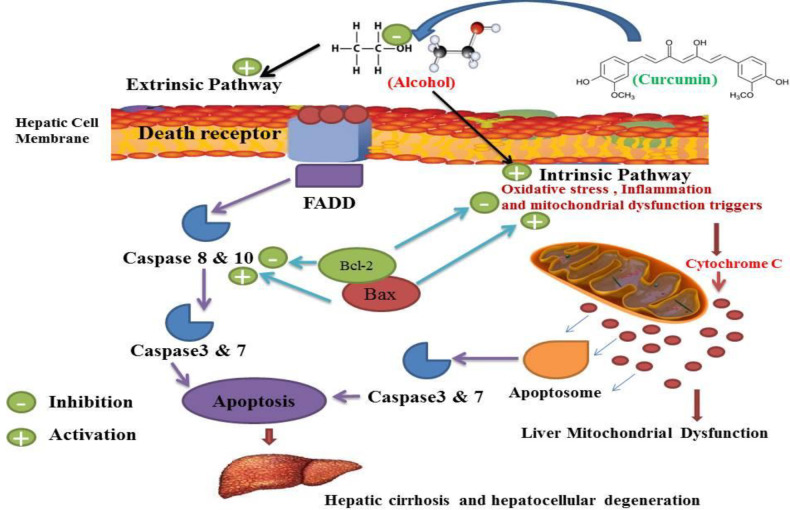
Curcumin may inhibit alcohol-induced apoptosis by inhibiting intestinal and extrinsic apoptosis, which may inhibit alcohol-induced hepatocellular degeneration and cirrhosis. Alcohol in the extrinsic pathway of apoptosis can trigger death receptor and adaptor protein consequences such as activates FADD, leading to activation of caspase 8 and 10 and caspase 3 and 7 which lead to apoptosis occurrences. Alcohol also causes mitochondrial dysfunction (as indicated in Figure 7) and increased production of cytochrome c, which may lead to the activation of the intestinal apoptosis pathway leading to the activation of caspase 3 and 7 and apoptosis. The activation consequences of these two pathways are apoptosis which plays a critical role in hepatocellular degeneration and cirrhosis. Curcumin inhibited the occurrence of these two pathways. Bcl-2 acts as an anti-apoptotic agent and Bax acts as an apoptotic agent and modulates apoptotic mediators and mitochondrial functions

Concerning oxidative stress and alcohol-induced inflammation in other aspects of alcohol-induced liver sequels, our research has shown that alcohol can cause apoptosis (127, 128). Alcohol Liver Disease (ALD) that occurs after liver cell death is one of the most common types of chronic liver disease that can progress to end-stage liver disease (8, 128-130). ALD has been characterized by histopathology, ranging from simple fat accumulation in the liver (steatosis), steatosis plus inflammation, Mallory bodies, hepatocytes, advanced fibrosis and cirrhosis (54, 130-133). It would appear that mitochondrial dysfunction and reactive oxygen species development play an important role in the progression from simple steatosis to steatohepatitis in ALD (134, 135). The pathogenesis of ALD in relation to tissue death is still not fully understood (130). Long-term alcohol consumption is known to lead to steatosis, which can progress to steatohepatitis and cirrhosis (133, 136 and 137). Although changes in lipid metabolism and increased synthesis of fatty acids and reduction in mitochondrial ß-oxidation and alteration of the redox state responsible for ALD progression are less clear (133, 137-139). Many documents suggest that hepatocyte apoptosis is a key mechanism for liver damage caused by alcohol (139, 140). Previous studies have shown that apoptosis of the liver cells is a major pathological feature of ALD in both experimental and human (140, 141). Pervious observation confirmed the activation of caspase in hepatocytes in alcohol abusers (142). Alcohol has been shown to increase Bax as an apoptotic protein and decrease Bcl2 as an anti-apoptotic protein. (142-144). This confirms and shows that alcohol and tobacco abuse can trigger apoptosis and eventually lead to brain cell damage and death (144). Alcohol abuse can cause liver damage by activating multiple apoptotic cascades and can result in hepatocyte DNA damage responsible for liver cell death and enzymatic and cell-key protein production disorder (145, 146). The magnitude of hepatocyte apoptosis was shown to be correlated with the severity of alcohol abuse (146, 147). This hepatocyte apoptosis was confirmed with increased scaling of bilirubin, alanine aminotransferase (ALT) and aspartate aminotransferase (AST) (147). The apoptotic index was observed in ALD patients and increased in liver specimens (148). In alcohol administration, apoptotic cell death with histological disasters has been observed and this phenomenon has been correlated with the degree of inflammation, oxidative stress and fibrosis stage (137, 149). Experimental studies have shown that exposure to alcohol results in increased cell death by both *in-vivo* and *in-vitro* apoptosis (149, 150). Abuse of alcohol has been shown to stimulate both the intrinsic and the extrinsic pathways (151, 152). Several mechanisms suggested that alcohol-mediated hepatocyte apoptosis by the intrinsic pathway was due to cellular and mitochondrial dysfunction caused by alcohol (152, 153). Injury caused by alcohol apoptosis was correlated with ROS production such as O2-and OH- (142, 153-155). Alcohol-induced mitochondrial dysfunction can lead to increased ROS production involving apoptosis induction (153). Alcohol-induced mitochondrial dysfunction can lead to increased ROS production involving apoptosis induction (10). Alcohol induced extrinsic apoptosis by activation of the induced death receptor was also shown, in particular by activation of Fas/Fas-ligand and TNF 1/TNF-a systems (145, 156). Since hepatocytes also expressed Fas receptor, the death of hepatocytes in alcoholic liver damage may occur through paracrine or autocrine mechanisms mediated by the hepatocytes themselves (10). Previous studies have shown that curcumin is an alcohol-protective agent and another hepatocellular apoptosis-induced agent is characterized by increased liver enzymes and lactate dehydrogenase activity (25, 157). Alcohol has also shown a decrease in total plasma protein, albumin and globulin, increased plasma bilirubin, while curcumin can control these types of alcohol induced malicious effects (113, 158, 159). The liver is the main source of serum proteins in which parenchymal cells are responsible for fibrinogen synthesis, albumin, many coagulation factors, and most globulins (160). Previous studies have shown that the use of curcumin affected significant protection against alcohol-induced hepatic necrosis (32, 161). Previous work showed hepatoprotective effect of curcumin against alcohol and similar agent induced liver damage (34). This agent is widely regarded as a therapeutic agent for hepatocellular diseases and events and can also be used for the management of hepatocellular related disorders (26, 69). Curcumin has possible hepato-protective enhancer properties in both animal and human subjects (26, 33). Curcumin antioxidant and anti-inflammatory behavior has been approved in previous studies (31, 33). Curcumin therapy has been shown to counteract apoptosis and reduce the increase in apoptotic biomarkers in liver damage events, especially after alcohol abuse (86, 162). All of these properties may contribute to the therapeutic potential efficacy of curcumin in hepatocellular disorders of alcohol abuse, but its exact mechanism remains unclear (32, 72). Previous results demonstrated the anti-apoptotic effect of curcumin against Bax reduction and improved Bcl-2 expressions in the liver (81, 162). These studies have shown that curcumin therapy attenuates the cleaved caspase-3 and the production of Bax and nuclear condensation resulting from some hepatocellular disorders by alcohol abusers (163, 164). In fact, curcumin has been shown to inhibit cell death by inhibiting DNA fragmentation in the apoptosis process during hepatocellular disorders of process alcohol abuse (164). Such studies have strongly suggested the liability of curcumin’s anti-apoptosis activity for the approved effects of alcohol abuse (32, 164). Curcumin controlled and normalized the process of cell cycle during normal regeneration of the liver by alcohol abuse (70, 165). Several studies on hepato-protective properties of curcumin, suggesting anti-apoptosis effects of curcumin, have been mediated by management of both the intestinal and the extrinsic pathways (Figure 8) (33, 165). Various mechanisms of action for curcumin protection against oxidative, inflammatory and anti-apoptosis have been described which mitochondrial dysfunction is the most important (25, 32, 70 and 166). Previous studies have shown that curcumin inhibits apoptosis in alcohol-induced liver cell damage in animals and this mechanism can inhibit hepatocellular cell death (25, 32 and 167).

**Figure 9:**
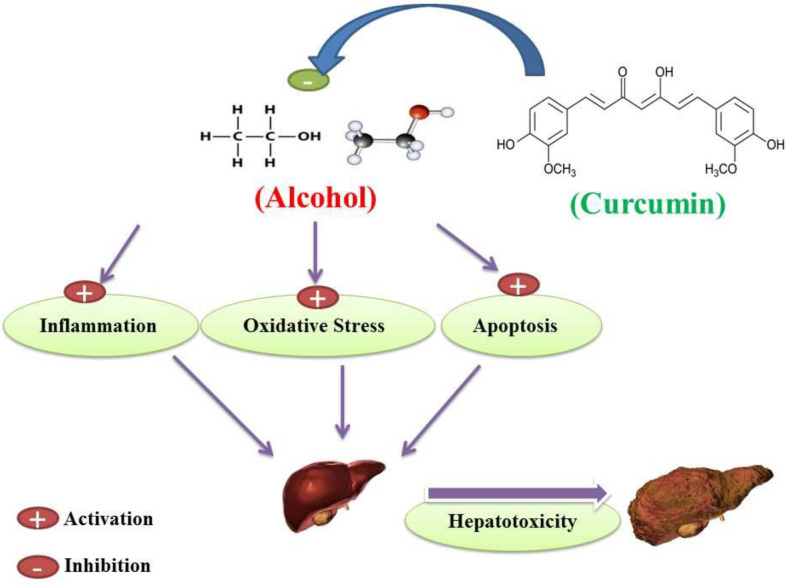
A whole brief history of curcumin heptoprotective effects against alcohol-induced hepatotoxicity. Curcumin inhibition of alcohol-induced oxidative stress, inflammation and apoptosis may protect hepatocellular tissue in alcohol-induced subjects and prevent the occurrence of alcohol-induced hepatotoxicity

## Conclusion

According to the mentioned side effects of alcohol abuse on hepatocellular denegation and cirrhosis induction as a result of the literature review, it has been shown that alcohol abuse can cause mitochondrial dysfunction in hepatocytes and also cause induction of lipid peroxidation and glutathione circulatory disorders as well as antioxidant enzymes. Alcohol-induced mitochondrial dysfunction in hepatocytes may also initiate inflammation and increase the production of pro-inflammatory biomarkers that lead to hepatocyte cell dysfunction. According to current literature, alcohol-induced liver cell cirrhosis has been mediated by apoptosis in both intrinsic and extrinsic pathways. According to the various advantages of curcumin results from the current literature review, curcumin may act as a potent hepato-protective agent in liver dysfunction in alcohol abuse subjects. Based on reported literature, curcumin has been shown to inhibit alcohol-induced mitochondrial dysfunction, lipid peroxidation, antioxidant enzymes, and glutathione ring defect. Literature indicates that curcumin may inhibit inflammation in hepatic alcohol abuser cells. According to the results of studies reviewed in the current literature, curcumin may inhibit alcohol-induced mitochondrial dysfunction and thus prevent the occurrence of apoptosis. On the other hand, curcumin inhibition of a cell death signaling pathway that activates by alcohol in hepatocellular tissue may inhibit the occurrence of an extrinsic pathway of apoptosis. Taken together, according to the type of literature, curcumin treatment of alcohol abusers in both human and animal subjects may reduce alcohol-induced hepatocellular apoptosis, oxidative stress and inflammation, and may act as a hepato-protective agent against alcohol-induced hepatotoxicity (Figure 9).
